# Neuromuscular anatomy of common fibular nerve with special focus on fibularis tertius muscle

**DOI:** 10.1007/s12565-025-00851-4

**Published:** 2025-05-18

**Authors:** Yutaro Natsuyama, Shuang-Qin Yi, Shinichi Kawata, Tomiko Yakura, Zhong-Lian Li, Hidenobu Miyaso, Masahiro Itoh

**Affiliations:** 1 Department of Anatomy, Tokyo Medical University, 6-1-1, Shinjuku, Tokyo, Japan; 2 Department of Frontier Health Sciences, Graduate School of Human Health Sciences, Tokyo Metropolitan University, Tokyo, 116-8551 Japan

**Keywords:** Common fibular nerve, Fibularis tertius muscle, Extensor digitorum longus muscle, Modified Shiler’s stain

## Abstract

Fibularis tertius muscle (FT) was first described by Vesalius in 1543 and was subsequently studied in detail by Henle and Hyrtl in the nineteenth century. There has been a controversy over whether FT is a separate beings. With regard to the question, it has been reported that the FT is an independent muscle or a part of the adjacent muscles such as the extensor digitorum longus muscle (EDL) and the extensor digitorum brevis muscle. In this study, to clarify the origin of the FT from the perspective of neuromuscular anatomy, gross and stereomicroscopic dissections and modified Shiler’s staining were performed on 48 sides of the lower legs from 25 Japanese cadavers. Of the 48 specimens, 43 specimens (89.6%) had a FT muscle belly, while 5 specimens (10.4%) did not. Most FTs were innervated by distal nerve branches from a common trunk with the EDL. The innervations of the FT and EDL suggest that the FT and EDL were brother muscles, and we proposed here a hypothesis that the FT and EDL originate from a common muscle mass and compete in a tug-of-war for muscle belly components during their development.

**Supplementary Information:**

The online version contains supplementary material available at 10.1007/s12565-025-00851-4.

## Introduction

In humans, the fibularis tertius muscle (FT) originates from the distal fibula, courses caudally to the extensor digitorum longus muscle (EDL), and attaches at the fourth and fifth metatarsal bones (Krammer et Al. 1979; Ercikti et Al. 2016; Olewnik et al. 2019). The FT was first described by Vesalius (1543) and was subsequently studied in detail by Henle (1858) and Hyrtl (1862) in the nineteenth century.

There has been a controversy over whether FT is a separate one or a part of the adjacent muscles (Yammine and Erić 2017). With regard to this issue, it has been reported that the FT is part of the EDL (Braus and ELze 1958; Gray and Basmajian 1968; Yammine and Erić 2017) or the extensor digitorum brevis muscle (Jones 2001). Other studies reported that FT is an independent one (Krammer et al. 1979; Yammine and Erić 2017). These reports focused on the morphology of muscles and tendons of the FT but did not discuss a relationship to their innervation.

In general, the basis for the relationship between nerves and muscles has been summarized in three laws (Shinohara 1996). The first is the “law of migration”. This law means that the innervation is an indicator for muscle migrated. This is because the nerve is attached to the muscle mass in the early development. The second is “law of fusion”. It means that the innervation is an indicator of muscle fusion. Since the nerves already connected to muscles in the early stages of development, when the two muscles fuse in the later stages, a muscle is formed that is innervated by two different types of nerves. The third is “law of separation”. This law means that the innervation is an indicator of muscle separation. Two different muscles supplied by one nerve are considered to originate from a single muscle mass. This type of muscle is called a "brother muscle". For example, the trapezius and sternocleidomastoid muscles are brother muscles. Studies on the relationship between nerves and muscles were reported in other structure (Koizumi 2022; Sakuraya et al. 2023).

In the present study, we aimed to clarify the origin of the FT from the perspective of the neuromuscular anatomy in human.

## Materials and methods

A total of 48 sides (24 right and 24 left) from 25 Japanese cadavers (22 males and 23 females; mean age at death, 88.3 years; range, 70–104 years) were used. All specimens had no history of orthopedic problems in the lower limbs. Study approval was obtained from the Ethics Committee of Tokyo Medical University (Approval Number: T2020-0050). All cadavers were fixed by arterial perfusion of 3.8% formalin and preserved in 60–70% alcohol.

First, morphological parameters of the FT were measured in order to classify the FT according to the length of the FT muscle belly. As a result, 4 groups were prepared, and 4 samples were randomly selected from each group for detailed analysis of the nerve distribution. Analysis of nerve distribution was performed using a stereomicroscope (OLYMPUS SZX7, Olympus, Tokyo, Japan) when necessary, and modified Shiler’s staining was additionally performed when necessary. The findings were photographed and illustrated using Adobe Illustrator 2024 (Adobe Inc., USA).

### Dissection procedure and measurements

After removing the skin from the lower limb, the lengths of the FT belly and the fibula, and the height of the FT origin were measured and the length ratio of the FT belly to the fibula was calculated. The length of the FT belly was measured from its cranial origin to the caudal origin. The length of the fibula was measured from its cranial to the caudal epiphysis of the fibula. The height of the FT origin was measured from the caudal epiphysis of the fibula to the cranial FT origin. The length ratio of the FT belly to the fibula was calculated by the length of the FT belly/the length of the fibula × 100(%).

The length of the FT belly was 79.2 ± 42.9 cm (mean ± standard deviation). Due to the large standard deviation, it was arbitrarily divided into following 4 groups based on the length of the FT belly (Table 1); Group 1: Large FT (the length of the Group 1 FT belly≥ 120 mm), Group 2: Intermediate FT (120 mm > the length of the Group 2 FT belly ≥ 50 mm), Group 3: Small FT (50 mm > the length of the Group 3 FT belly > 0 mm), Group 4: Lacked FT belly (the length of the Group 4 FT belly = 0 mm). Four specimens were randomly selected from each group, and the common fibular nerve and its innervated muscles were sampled as a single unit and the nerve branching patterns were analysed in detail.

**Table 1:** Morphological variation of fibularis tertius muscle (FT) in humans

Parameter	Group 1 n = 7 (14.6%)	Group 2 n = 27 (56.3%)	Group 3 n = 9 (18.8%)	Group 4 n = 5 (10.4%)	Total n = 48
Length of the FT belly (mm)	141.7±10.1^a,c^	89.4±14.9^a,b^	44.1±31.8^b,c^		79.2±42.9
Length of the fibula (mm)	350.3±20.1	331.5±20.9	332.7±21.6	340.6±21.7	335.4±22.1
Length ratio of the FT belly to the fibula (%)	40.55±3.3^a,c^	27.1±4.9^a,b^	13.5±10.1^b,c^		23.7±12.5
Height of the FT origin (mm)	218.6±20.1^a,c^	165.7±22.9^a,b^	131.3±29.7^b,c^		147.2±64.3
age (years)	86.0±6.7	88.2±8.3	86.3±9.3	92.2±2.6	88.3±8.1

Parameters are shown as the mean ± standard deviation of the date from each group.

Group 1: Large FT (the length of the Group 1 FT belly≥ 120 mm), Group 2: Intermediate FT (120 mm > the length of the Group 2 FT belly ≥ 50 mm), Group 3: Small FT (50 mm > the length of the Group 3 FT belly > 0 mm), Group 4: Lacked FT belly (the length of the Group 4 FT belly = 0 mm).

^a^*p* < 0.001 Group 1 versus Group 2; ^b^*p* < 0.001 Group 2 versus Group 3; ^a^*p* < 0.001 Group 1 versus Group 3

### Analysis of the nerve branching patterns of nerve fascicles

Neuromuscular specimens were dissected by peeling off the epineuria until the nerve reached each muscle. These dissections were performed using a stereomicroscope (OLYMPUS SZX7, Olympus, Tokyo, Japan) if necessary, and modifies Shiler’s staining was also performed if necessary.

### Modified Sihler’s staining

A modified Sihler staining was used to visualize the distribution of intramuscular nerve supply in the FT (Liu et al. 1997; Sekiya et al. 2005). The method is described briefly as follows:

1. Maceration and depigmentation (four weeks): 3% aqueous potassium hydroxide (KOH) solution add 0.2 mL of 3% hydrogen peroxide to 100 mL 3% KOH solution for depigmentation;
2. Decalcification (eight days): Sihler’s solution I: one volume of glacial acetic acid, two volumes of glycerin, and twelve volumes of 1% aqueous chloral hydrate;
3. Staining (two weeks): Sihler’s solution II: one volume stock Ehrlich’s hematoxylin, two volumes of glycerin, and twelve volumes of 1% aqueous chloral hydrate;
4. Destaining (three hours): Sihler’s solution I;
5. Neutralization (three hours): 0.05% lithium carbonate solution;
6. Clearing (three days each): Aqueous glycerin 50% and 100% glycerin.

### Statistical analysis

The mean and standard deviation were calculated for all of the data quantified in this study. F-tests and T-tests were performed at a significance level of 0.01 for the data on age, the length of fibula for each of Groups 1–4, and the height of the FT origin, the length of the FT belly, and the length ratio of the FT belly to the fibula for each of Groups 1–3 (Table 1).

## Results

Of the 48 specimens, 43 specimens (89.6 %) were observed to have the FT belly (Figures 1, 2, 3, 4 and 5, Supplementary Figs. 1–7), while 5 specimens (10.4 %) did not (Figure 6, Supplementary Figs. 8–10). Furthermore, intertendonous connections between the FT and EDL were found in 5 of the 43 specimens, as shown in Fig. 3. However, no intertendonous connection was observed between the FT and extensor digitorum brevis muscle in all specimens. There were no significant differences in age and the length of the fibula between Groups 1–4 (Table 1).

**Fig. 1:**
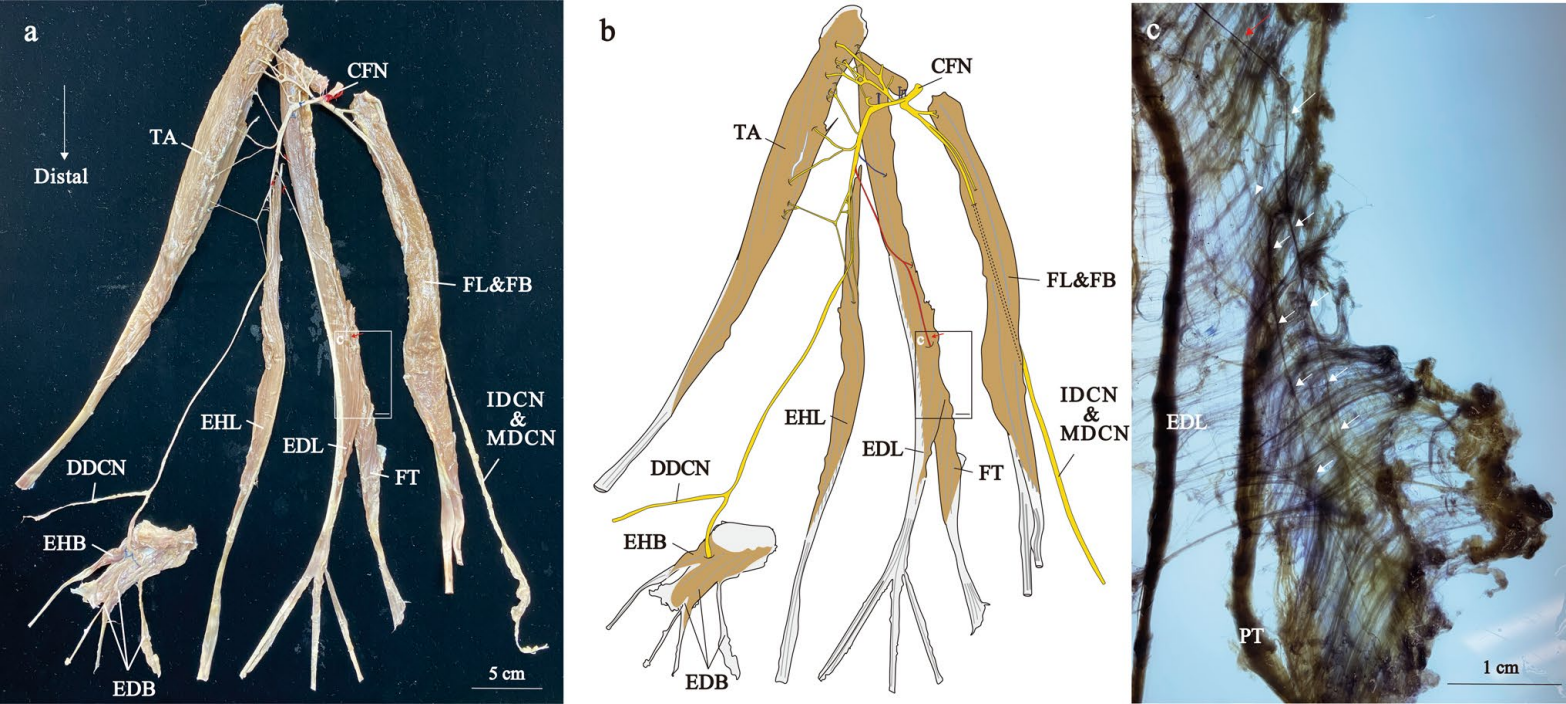
The distribution of the right common fibular nerve in Group 1. Photograph (**a**) and illustration (**b**) show neuromuscular unit. Enlarged views of the square frames within (**a**, **b**) and the modified Shiler's stain are shown (**c**). Red arrows indicate the point where the nerve starts to distribute to the EDL, white arrows indicate the nerve branch to the FT and white arrowhead indicates the nerve distributing to the EDL. CFN, common fibular nerve; DDCN, dorsal digital nerve; EDB, extensor digitorum brevis muscle; EDL, extensor digitorum longus muscle; EHB, extensor hallucis brevis muscle; EHL, extensor hallucis longus muscle; FB, fibularis brevis muscle; FL, fibularis longus muscle; FT, fibularis tertius muscle; IDCN, intermediate dorsal cutaneous nerve; MDCN, medial dorsal cutaneous nerve; TA, tibialis anterior muscle

**Fig. 2:**
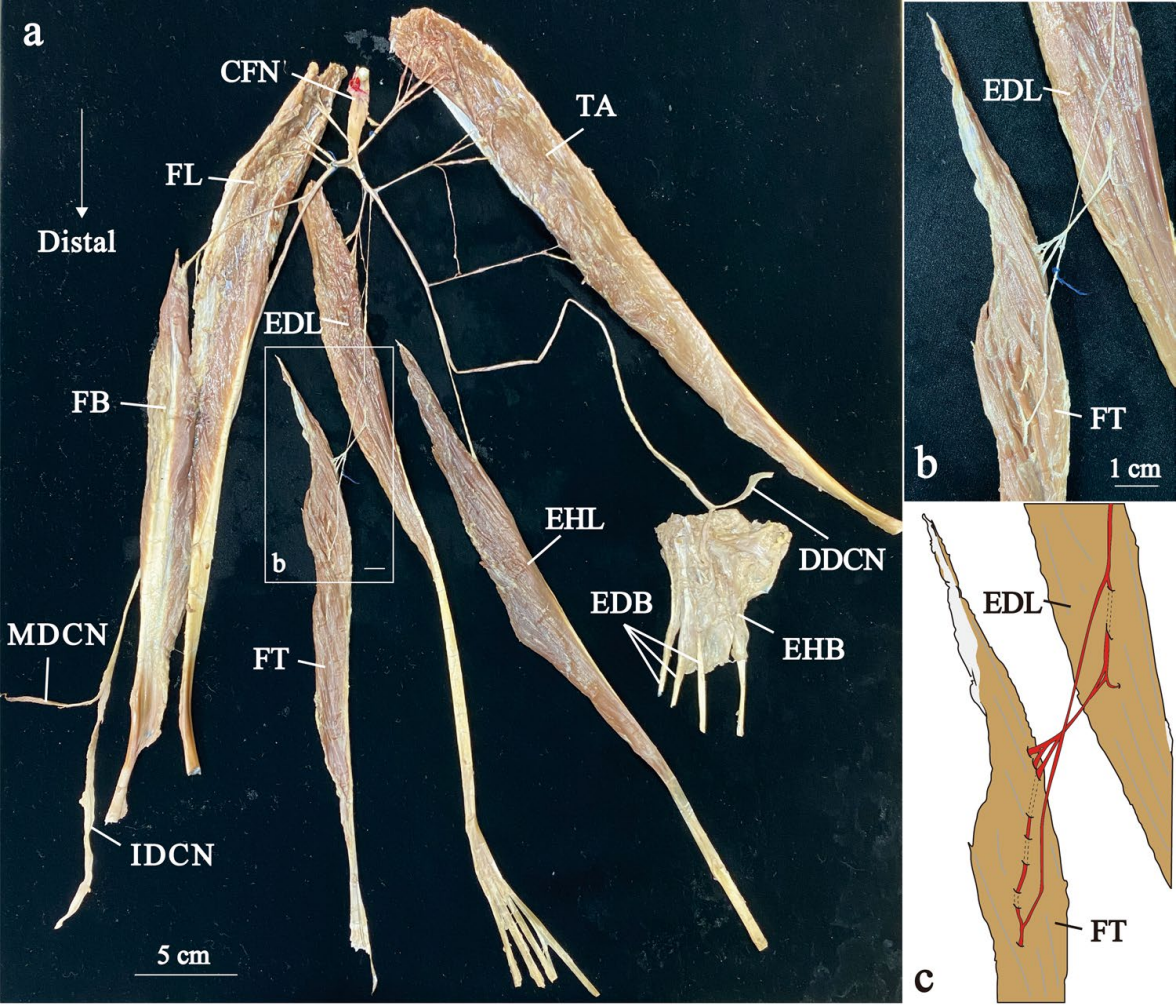
The distribution of the left common fibular nerve in Group 1. Photograph (**a**) and enlarged views of the square frames within a (**b**) show neuromuscular unit. Illustration of the b (**c**). Distal nerve branches are painted red. CFN, common fibular nerve; DDCN, dorsal digital nerve; EDB, extensor digitorum brevis muscle; EDL, extensor digitorum longus muscle; EHB, extensor hallucis brevis muscle; EHL, extensor hallucis longus muscle; FB, fibularis brevis muscle; FL, fibularis longus muscle; FT, fibularis tertius muscle; IDCN, intermediate dorsal cutaneous nerve; MDCN, medial dorsal cutaneous nerve; TA, tibialis anterior muscle

**Fig. 3:**
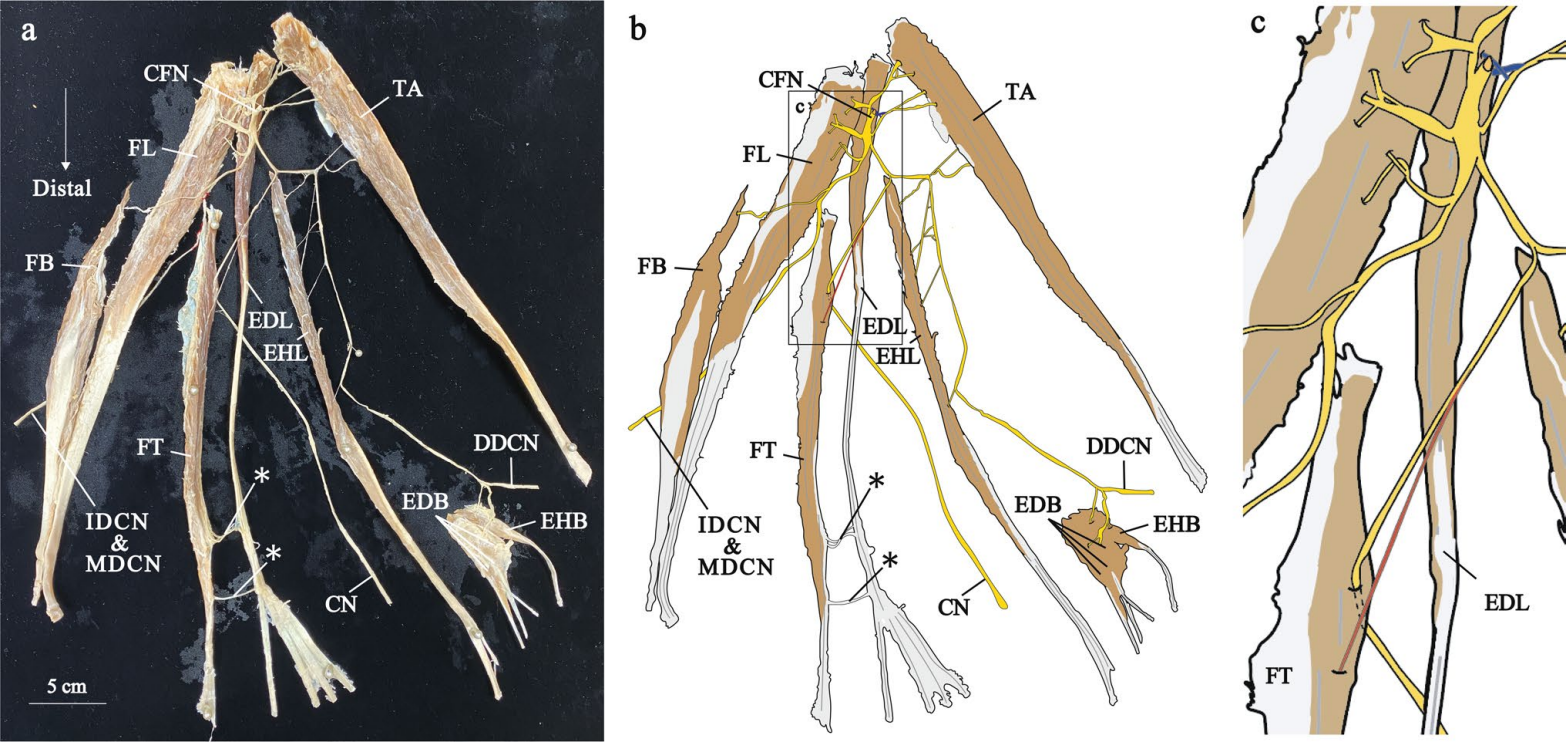
The distribution of the left common fibular nerve in Group 1. This specimen was the largest FT belly (150.7 mm) in the study. Photograph (**a**) and illustration (**b**) show neuromuscular unit. Proximal nerve branches and distal nerve branch are painted blue and red, respectively. Enlarged views of the square frames within b (**c**). CFN, common fibular nerve; CN, cutaneous nerve; DDCN, dorsal digital nerve; EDB, extensor digitorum brevis muscle; EDL, extensor digitorum longus muscle; EHB, extensor hallucis brevis muscle; EHL, extensor hallucis longus muscle; FB, fibularis brevis muscle; FL, fibularis longus muscle; FT, fibularis tertius muscle; IDCN, intermediate dorsal cutaneous nerve; MDCN, medial dorsal cutaneous nerve; TA, tibialis anterior muscle

**Fig. 4:**
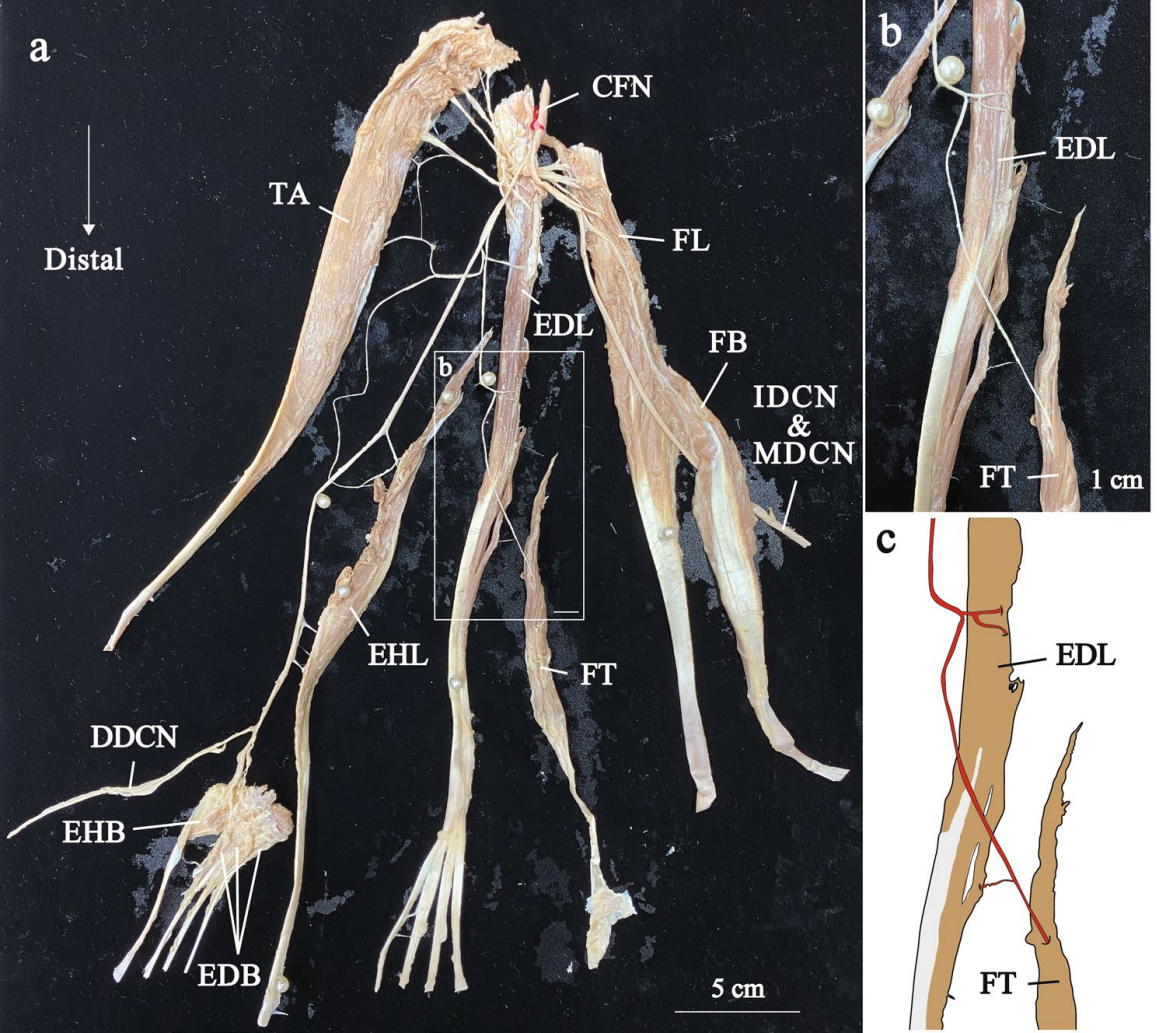
The distribution of the right common fibular nerve in Group 2. Photograph (**a**) and enlarged views of the square frames within a (**b**) show neuromuscular unit. Illustration of the b (**c**). Distal nerve branches are painted red. CFN, common fibular nerve; DDCN, dorsal digital nerve; EDB, extensor digitorum brevis muscle; EDL, extensor digitorum longus muscle; EHB, extensor hallucis brevis muscle; EHL, extensor hallucis longus muscle; FB, fibularis brevis muscle; FL, fibularis longus muscle; FT, fibularis tertius muscle; IDCN, intermediate dorsal cutaneous nerve; MDCN, medial dorsal cutaneous nerve; TA, tibialis anterior muscle

**Fig. 5:**
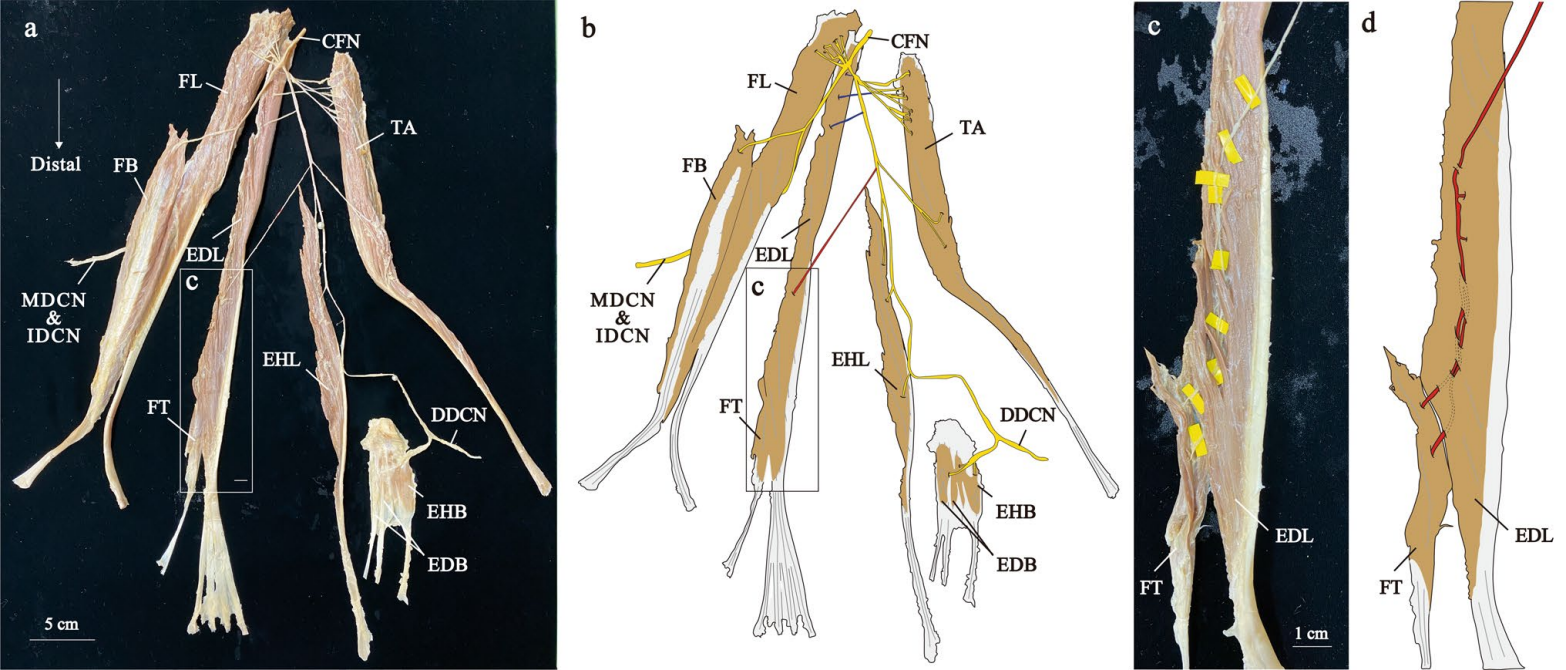
The distribution of the left common fibular nerve in Group 3. Photograph (**a**) and illustration (**b**) show neuromuscular unit. Proximal nerve branches and distal nerve branches are painted blue and red, respectively. Enlarged views of the square frames within (**a**, **b**) and the proceeded dissection are shown (**c**). Illustration of the c (**d**). CFN, common fibular nerve; DDCN, dorsal digital nerve; EDB, extensor digitorum brevis muscle; EDL, extensor digitorum longus muscle; EHB, extensor hallucis brevis muscle; EHL, extensor hallucis longus muscle; FB, fibularis brevis muscle; FL, fibularis longus muscle; FT, fibularis tertius muscle; IDCN, intermediate dorsal cutaneous nerve; MDCN, medial dorsal cutaneous nerve; TA, tibialis anterior muscle

**Fig. 6:**
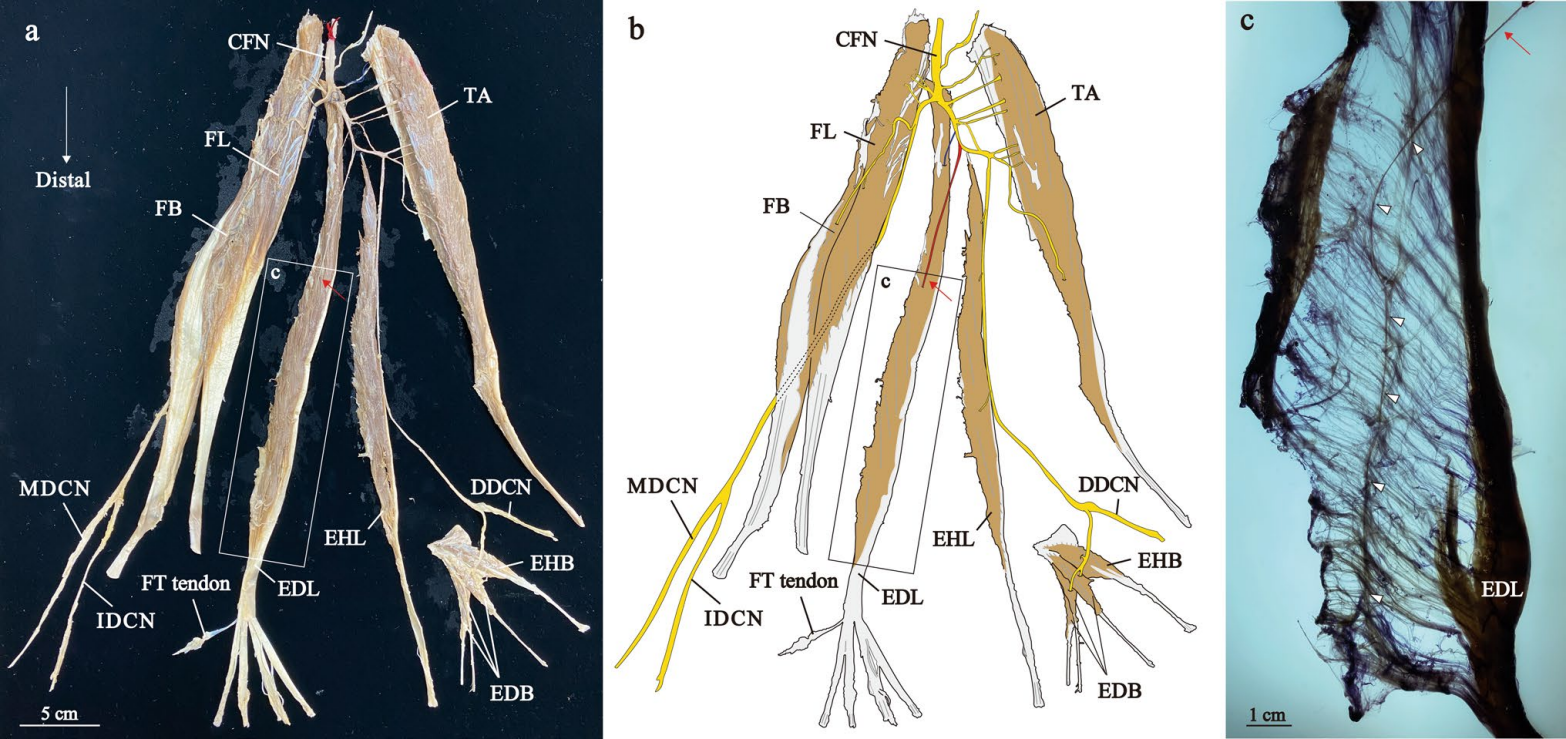
The distribution of the left common fibular nerve in Group 4. Photograph (**a**) and illustration (**b**) show neuromuscular unit. Proximal nerve branch and distal nerve branch are painted blue and red, respectively. Enlarged views of the square frames within (**a**, **b**) and the modified Shiler's stain are shown (**c**). Red arrows indicate the point where the nerve starts to distribute to the muscle, and arrowheads indicate the nerve distributing to the EDL. CFN, common fibular nerve; DDCN, dorsal digital nerve; EDB, extensor digitorum brevis muscle; EDL, extensor digitorum longus muscle; EHB, extensor hallucis brevis muscle; EHL, extensor hallucis longus muscle; FB, fibularis brevis muscle; FL, fibularis longus muscle; FT, fibularis tertius muscle; IDCN, intermediate dorsal cutaneous nerve; MDCN, medial dorsal cutaneous nerve; TA, tibialis anterior muscle

**Fig. 7:**
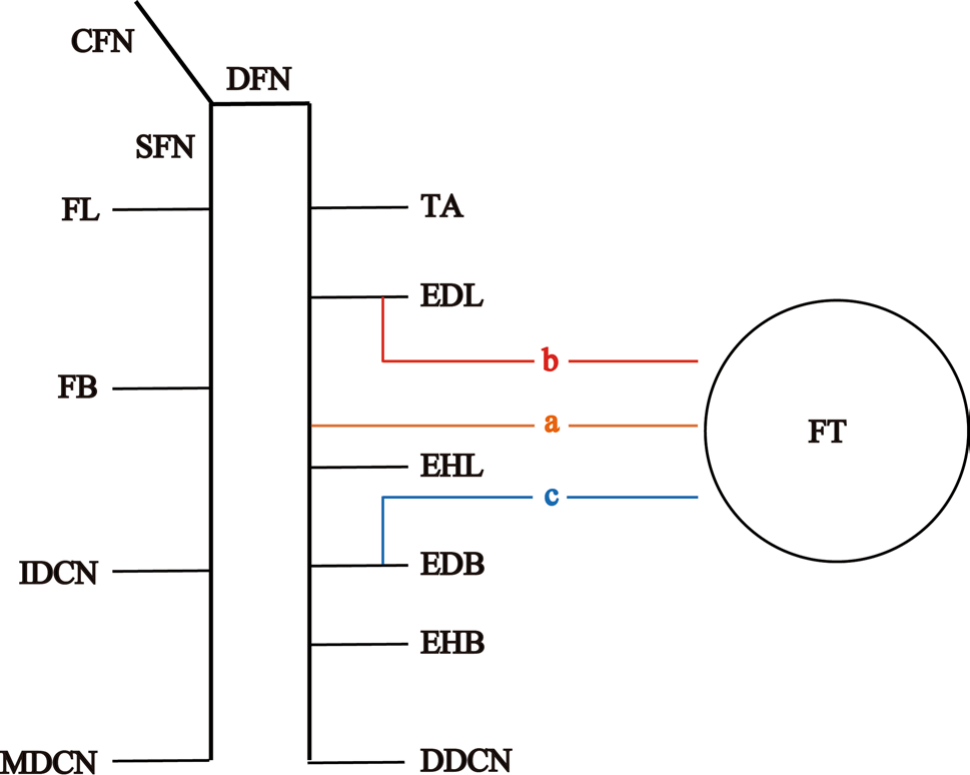
Hypothetical diagram of the nerve distribution to the fibularis tertius muscle. **a** separate nerve to the FT; **b** nerve to the FT from common trunk with the EDL; **c** nerve to the FT from common trunk with the EDB; CFN, common fibular nerve; DDCN, dorsal digital nerve; DFN, deep fibular nerve; EDB, extensor digitorum brevis muscle; EDL, extensor digitorum longus muscle; EHB, extensor hallucis brevis muscle; EHL, extensor hallucis longus muscle; FB, fibularis brevis muscle; FL, fibularis longus muscle; FT, fibularis tertius muscle; IDCN, intermediate dorsal cutaneous nerve; MDCN, medial dorsal cutaneous nerve; SFN, superficial fibular nerve; TA, tibialis anterior muscle

Our hypothesis is as follows; if the nerve innervates the FT independently (“a” in Fig. 7), the FT is considered to be an independent muscle; if the nerve innervates the FT with the common trunk of the nerve branch of the EDL (“b” in Fig. 7), the FT and EDL are considered to be brother muscles; if the nerve innervates the FT with a common trunk of the nerve branch of the extensor digitorum brevis muscle (“c” in Fig. 7), these 2 muscles are considered to be brother muscles.

### Group 1

In Group 1 that has a large FT belly, the height of the FT origin and the length ratio of the FT belly to the fibula were significantly greater than those in Groups 2 and 3 (Table 1). In contrast, the EDL belly was relatively small compared with Groups 2 and 3.

Nerve branches to the FT mainly branched from the common trunk with the EDL nerve branching from middle region of the EDL belly (Fig. 1b, the nerve painted red). This branch was called the distal nerve branch. In contrast, many nerve branches to the EDL were observed in the proximal part of the EDL (Fig. 1b, the nerve painted blue). These branches were called the proximal nerve branches. The common fibular nerve was divided into the deep and superficial fibular nerves at the lower part of the fibular head, and there were many communicating branches in the proximal part and fewer communicating branches in the distal part. Using modified Shiler’s staining, we observed that the distal nerve branches coursed within the EDL eventually distributed to the FT, accompanied by sending out branches to the EDL (Fig. 1). Indeed, by carefully separating the muscle fibers, it was possible to observe distal nerve branches to the FT, which also innervated the EDL (Fig. 2). The largest FT belly (150.7 mm) was innervated separately with no common trunk with the EDL (Fig. 3). The remained specimen was also innervated by distal nerve branch from the common trunk with the EDL as described above (Supplementary Fig. 1).

### Groups 2 and 3

In Group 2 that has a FT belly of intermediate size, the height of the FT origin and the length ratio of the FT belly to the fibula were significantly greater than those in Group 3 that has a small FT belly (Table 1). As the FT belly became smaller, the EDL belly became bigger (Figs. 4 and 5).

The pattern of nerve branching was similar to that of Group 1 (Figs. 5 and 6, Supplementary Figs. 2–7). As in most specimens of Group 1, there were distal and proximal nerve branches, and the FT was innervated by the distal nerve branch, which arises from the common trunk with the nerve branch of the EDL (Figs. 4 and 5, Supplementary Figs. 2–7).

### Group 4

In Group 4 with no FT belly, the EDL belly was larger than Groups 1–3 (Figure 7, Supplementary Figs. 8–10).

The pattern of nerve branching was similar to that of Groups 1–3 (Fig. 6, Supplementary Figs. 8–10). Distal and proximal nerve branches were similarly observed, although there was no FT belly. From the middle region of the EDL belly, the distal nerve branch ran intramuscular of the EDL and reached caudal part of the EDL (Fig. 6, Supplementary Figs. 8–10).

## Discussion

The aim of the present study was to determine the origin of the FT from the perspective of the nerve innervation. The results showed that the majority of the FT were innervated by distal nerve branch from the common trunk with the EDL. Therefore, the FT and EDL were considered to be brother muscles based on the law of separation.

**Fig. 8:**
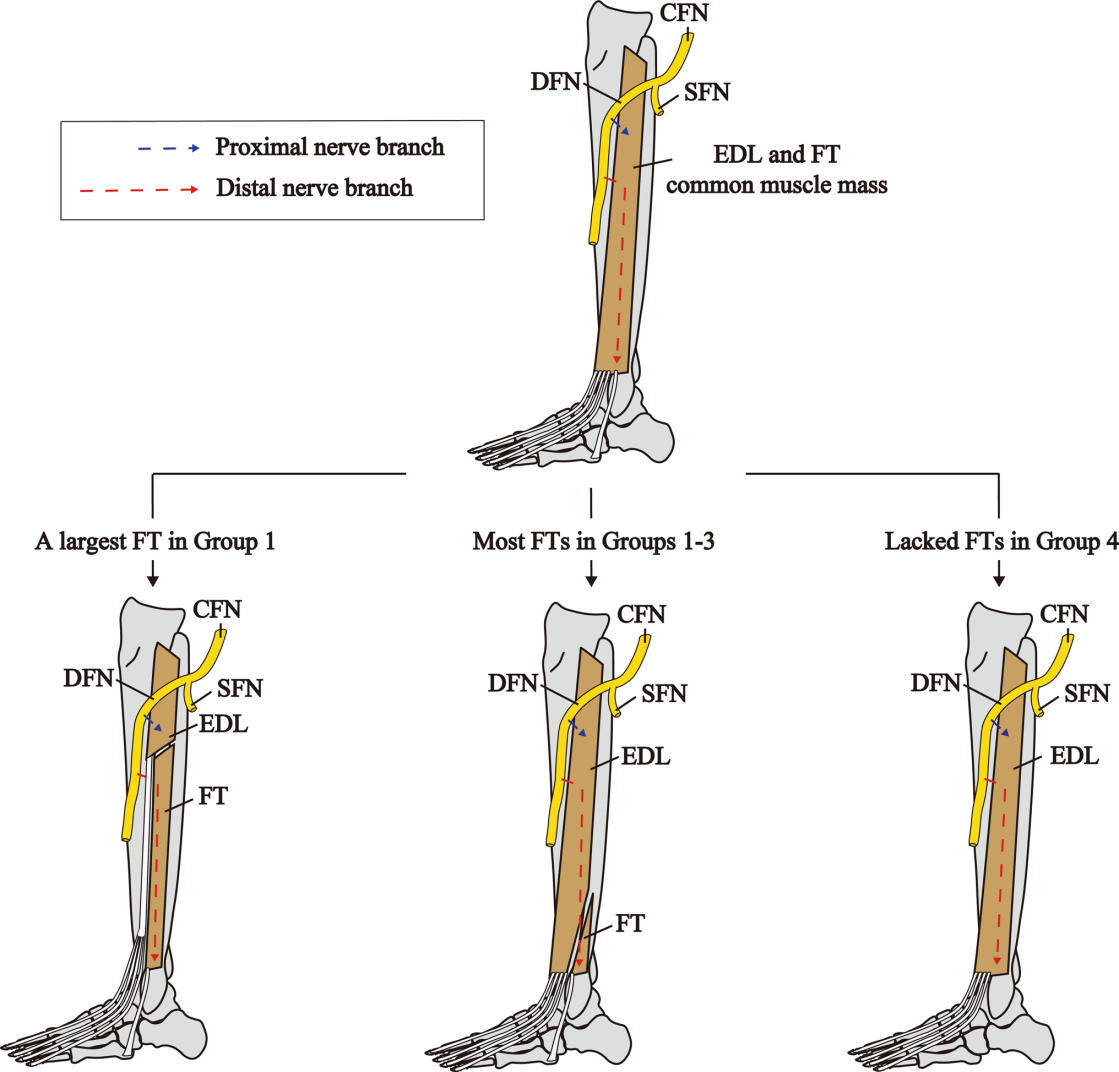
Hypothetical diagram of the FT and the EDL development. The top shows common muscle mass of the FT and the EDL, the bottom shows the mature muscles. Bottom from left to right shows the case of the largest FT, most FTs, and lacked FTs. Blue arrows and red arrows indicate proximal nerve branches and distal nerve branches, respectively. CFN, common fibular nerve; EDL, extensor digitorum longus muscle; FT, fibularis tertius muscle

In the present study, twelve specimens with the FT belly were dissected to reveal nerve branching pattern and the results showed that in eleven specimens were innervated by the distal nerve branch from the common trunk with the EDL. The largest FT belly was innervated separately, without a common trunk with the EDL (Fig. 4). This sample had the largest FT belly and the smallest EDL belly. In Group 4 without the FT belly, the distal nerve branches to the EDL also started to distribute at middle region of the EDL belly and distributed to the lower end. Based on the above, the nerve branches to the EDL and FT can be divided into two main parts: proximal and short nerve branch (proximal nerve branch, Fig. 8 blue) and distal and long nerve branch (distal nerve branch, Fig. 8 red). In this view, we proposed a hypothesis that EDL and FT originate from a common muscle mass and compete in like a tug-of-war for muscle belly components during development (Fig. 8). This hypothesis could explain the different innervation patterns and FT sizes; most FT was present distal to the middle of the lower leg, so the distal nerve branch innervated both the EDL and the FT; the largest FT was present cranial to the middle of the lower leg, so the distal nerve branch innervated only the FT; in the lacked FT, the distal nerve branch innervated the EDL to the lower end (Fig. 8). Intertendinous connections between the FT and the EDL were observed in 10.4% (5/48 specimens), and no intertendinous connections between the FT and the extensor digitorum brevis muscle were observed in all specimens. This information may also suggest a similarity between the FT and the EDL in perspective of the morphology. Although previous studies have reported the different heights of the FT origin (Joshi et al. 2006; Yammine and Erić 2017; Olewnik 2019), our hypothesis may explain these differences. As the height of the FT origin was synonymous with the length of the FT belly, and the length of the FT belly can be explained.

The FT is particularly developed in pigs (König and Liebich 2020; Natsuyama et al. 2024) and horses (Raes et al. 2011; König and Liebich 2020), where it differs in form that in humans. In pigs and horses, the FT has a common origin with the EDL, which originates from the anterior surface of the femur. We studied the pig FT with a focus on nerve distribution and found that nerves to the FT are distributed from a common trunk with the nerves to the EDL (Natsuyama et al. 2024). These results are similar to those of the present study and support that the FT and EDL are brother muscles. However, species differences may be present in regard to FT morphology; the FT consistently originates from the femur in pigs and horses, whereas there is a diversity of the FT forms in humans as shown in Groups 1–4, although the pattern of innervation of the FT is similar.

## Limitations

The present study is limited by the number bias of the samples. A total of 48 specimens were observed in the present study, with a lack rate of 10.4% in the FT belly, similar to previous studies (Joshi et al. 2006; Witvrouw et al. 2006; Rourke et al. 2007; Ercikti et al. 2016; Albay and Candan 2017; Yammine and Erić 2017). A total of 16 detailed nerve distributions were also observed, but further analysis would provide more robust data. However, no major differences in nerve branchial pattern were observed in the 16 samples, by which we suppose that the FT and EDL are brother muscle in general.

## Conclusions

In conclusion, nerve distributions of the FT and EDL suggest that the FT and EDL were brother muscles, and we proposed a hypothesis that the FT and the EDT originate from a common muscle mass and compete in like a tug-of-war for muscle belly components during development.

## Supplementary Information

Below is the link to the electronic supplementary material.

Supplementary file1 (DOCX 12 kb)

Supplementary file2 (JPG 20550 kb)

Supplementary file3 (JPG 19955 kb)

Supplementary file4 (JPG 23051 kb)

Supplementary file5 (JPG 18834 kb)

Supplementary file6 (JPG 18279 kb)

Supplementary file7 (JPG 21704 kb)

Supplementary file8 (JPG 23288 kb)

Supplementary file9 (JPG 16881 kb)

Supplementary file10 (JPG 18935 kb)

Supplementary file11 (JPG 20120 kb)
