# Supplementary material for: Neuromuscular anatomy of common fibular nerve with special focus on fibularis tertius muscle
Source: Anat Sci Int. 2025 May 18;101(2):193–201. doi: 10.1007/s12565-025-00851-4 (PMC12987884; doi:10.1007/s12565-025-00851-4)
Supplement: Supplementary file 1 — Supplementary file1 (DOCX 12 kb) [file 12565_2025_851_MOESM1_ESM.docx]

Supplementary Figure 1
The distribution of the right common fibular nerve in Group 1. Photograph (a) and enlarged views of the square frames within a (b) show neuromuscular unit. Black arrows and white arrows indicate nerve branches to the EDL and the FT, respectively. CFN, common fibular nerve; DDCN, dorsal digital nerve; EDB, extensor digitorum brevis muscle; EDL, extensor digitorum longus muscle; EHB, extensor hallucis brevis muscle; EHL, extensor hallucis longus muscle; FB, fibularis brevis muscle; FL, fibularis longus muscle; FT, fibularis tertius muscle; IDCN, intermediate dorsal cutaneous nerve; MDCN, medial dorsal cutaneous nerve; TA, tibialis anterior muscle.

Supplementary Figure 2
The distribution of the left common fibular nerve in Group 2. Photograph (a) and enlarged views of the square frames within a (b) show neuromuscular unit. Black arrows and white arrow indicate nerve branches to the EDL and the FT, respectively. CFN, common fibular nerve; DDCN, dorsal digital nerve; EDB, extensor digitorum brevis muscle; EDL, extensor digitorum longus muscle; EHB, extensor hallucis brevis muscle; EHL, extensor hallucis longus muscle; FB, fibularis brevis muscle; FL, fibularis longus muscle; FT, fibularis tertius muscle; IDCN, intermediate dorsal cutaneous nerve; MDCN, medial dorsal cutaneous nerve; TA, tibialis anterior muscle.

Supplementary Figure 3
The distribution of the left common fibular nerve in Group 2. Photograph (a) and enlarged views of the square frames within a (b) show neuromuscular unit. White arrows indicate nerve branches to the FT, respectively. CFN, common fibular nerve; DDCN, dorsal digital nerve; EDB, extensor digitorum brevis muscle; EDL, extensor digitorum longus muscle; EHB, extensor hallucis brevis muscle; EHL, extensor hallucis longus muscle; FB, fibularis brevis muscle; FL, fibularis longus muscle; FT, fibularis tertius muscle; IDCN, intermediate dorsal cutaneous nerve; MDCN, medial dorsal cutaneous nerve; TA, tibialis anterior muscle.

Supplementary Figure 4
The distribution of the right common fibular nerve in Group 2. Photograph (a) and enlarged views of the square frames within a (b) show neuromuscular unit. Black arrow and white arrows indicate nerve branches to the EDL and the FT, respectively. CFN, common fibular nerve; DDCN, dorsal digital nerve; EDB, extensor digitorum brevis muscle; EDL, extensor digitorum longus muscle; EHB, extensor hallucis brevis muscle; EHL, extensor hallucis longus muscle; FB, fibularis brevis muscle; FL, fibularis longus muscle; FT, fibularis tertius muscle; IDCN, intermediate dorsal cutaneous nerve; MDCN, medial dorsal cutaneous nerve; TA, tibialis anterior muscle.

Supplementary Figure 5
The distribution of the left common fibular nerve in Group 3. Photograph (a) and enlarged views of the square frames within a (b) show neuromuscular unit. Black arrows and white arrows indicate nerve branches to the EDL and the FT, respectively. CFN, common fibular nerve; DDCN, dorsal digital nerve; EDB, extensor digitorum brevis muscle; EDL, extensor digitorum longus muscle; EHB, extensor hallucis brevis muscle; EHL, extensor hallucis longus muscle; FB, fibularis brevis muscle; FL, fibularis longus muscle; FT, fibularis tertius muscle; IDCN, intermediate dorsal cutaneous nerve; MDCN, medial dorsal cutaneous nerve; TA, tibialis anterior muscle.

Supplementary Figure 6
The distribution of the right common fibular nerve in Group 3. Photograph (a) and enlarged views of the square frames within a (b) show neuromuscular unit. White arrows indicate nerve branches to the FT, respectively. CFN, common fibular nerve; DDCN, dorsal digital nerve; EDB, extensor digitorum brevis muscle; EDL, extensor digitorum longus muscle; EHB, extensor hallucis brevis muscle; EHL, extensor hallucis longus muscle; FB, fibularis brevis muscle; FL, fibularis longus muscle; FT, fibularis tertius muscle; IDCN, intermediate dorsal cutaneous nerve; MDCN, medial dorsal cutaneous nerve; TA, tibialis anterior muscle.

Supplementary Figure 7
The distribution of the left common fibular nerve in Group 3. Photograph (a) and enlarged views of the square frames within a (b) show neuromuscular unit. Black arrow and white arrows indicate nerve branches to the EDL and the FT, respectively. CFN, common fibular nerve; DDCN, dorsal digital nerve; EDB, extensor digitorum brevis muscle; EDL, extensor digitorum longus muscle; EHB, extensor hallucis brevis muscle; EHL, extensor hallucis longus muscle; FB, fibularis brevis muscle; FL, fibularis longus muscle; FT, fibularis tertius muscle; IDCN, intermediate dorsal cutaneous nerve; MDCN, medial dorsal cutaneous nerve; TA, tibialis anterior muscle.

Supplementary Figure 8
The distribution of the right common fibular nerve in Group 4. Photograph (a) and enlarged views of the square frames within a (b) show neuromuscular unit. Black arrows indicate nerve branches to the EDL. CFN, common fibular nerve; DDCN, dorsal digital nerve; EDB, extensor digitorum brevis muscle; EDL, extensor digitorum longus muscle; EHB, extensor hallucis brevis muscle; EHL, extensor hallucis longus muscle; FB, fibularis brevis muscle; FL, fibularis longus muscle; IDCN, intermediate dorsal cutaneous nerve; MDCN, medial dorsal cutaneous nerve; TA, tibialis anterior muscle.

Supplementary Figure 9
The distribution of the right common fibular nerve in Group 4. Photograph (a) and enlarged views of the square frames within a (b) show neuromuscular unit. Black arrows indicate nerve branches to the EDL. CFN, common fibular nerve; DDCN, dorsal digital nerve; EDB, extensor digitorum brevis muscle; EDL, extensor digitorum longus muscle; EHB, extensor hallucis brevis muscle; EHL, extensor hallucis longus muscle; FB, fibularis brevis muscle; FL, fibularis longus muscle; IDCN, intermediate dorsal cutaneous nerve; MDCN, medial dorsal cutaneous nerve; TA, tibialis anterior muscle.

Supplementary Figure 10
The distribution of the left common fibular nerve in Group 4. Photograph (a) and enlarged views of the square frames within a (b) show neuromuscular unit. Black arrows indicate nerve branches to the EDL. CFN, common fibular nerve; DDCN, dorsal digital nerve; EDB, extensor digitorum brevis muscle; EDL, extensor digitorum longus muscle; EHB, extensor hallucis brevis muscle; EHL, extensor hallucis longus muscle; FB, fibularis brevis muscle; FL, fibularis longus muscle; IDCN, intermediate dorsal cutaneous nerve; MDCN, medial dorsal cutaneous nerve; TA, tibialis anterior muscle.
