# Supplementary figures and images for: Neuromuscular anatomy of common fibular nerve with special focus on fibularis tertius muscle
Source: Anat Sci Int. 2025 May 18;101(2):193–201. doi: 10.1007/s12565-025-00851-4 (PMC12987884; doi:10.1007/s12565-025-00851-4)

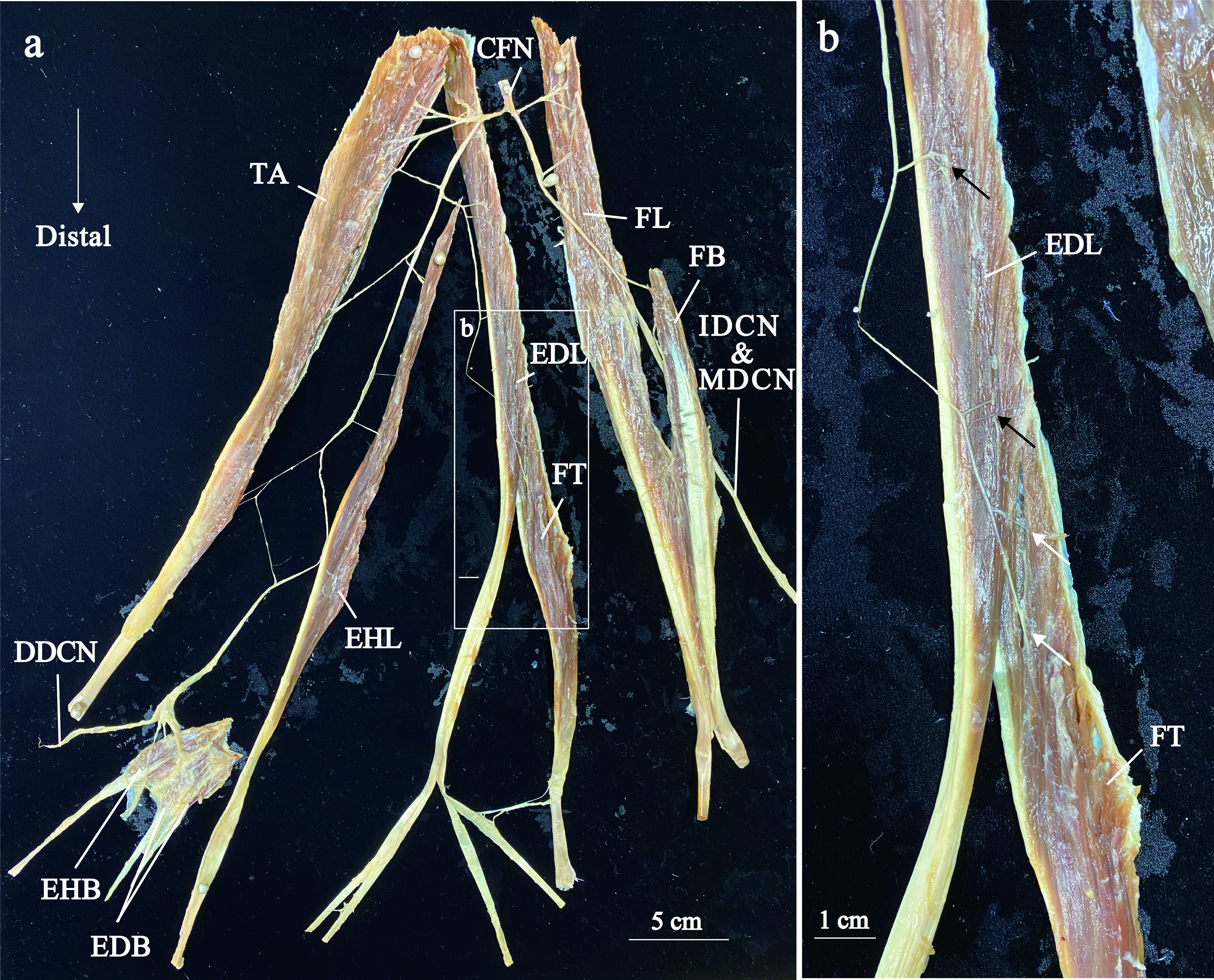

Supplement: Supplementary file 2 — Supplementary file2 (JPG 20550 kb) [file 12565_2025_851_MOESM2_ESM.jpg]

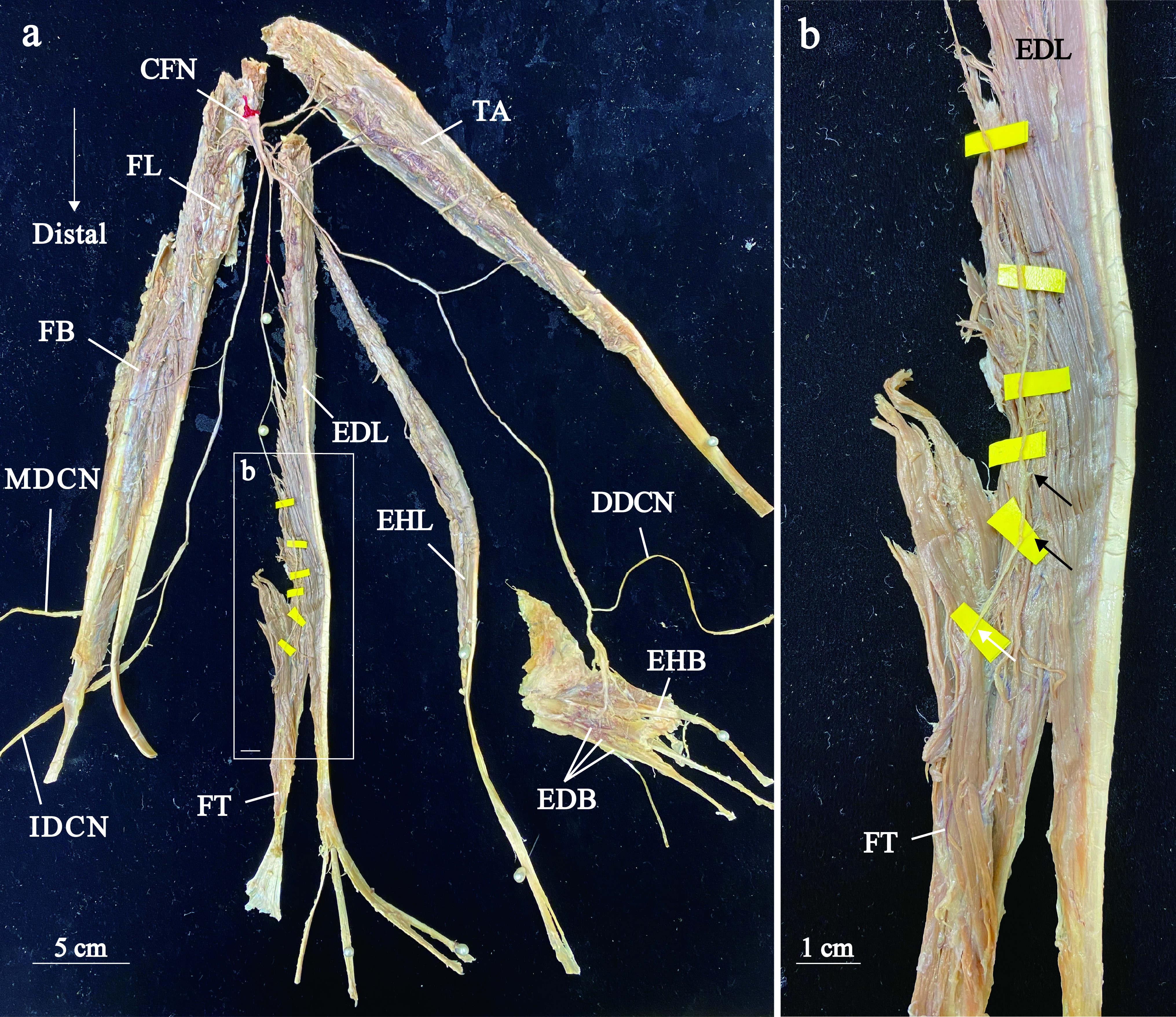

Supplement: Supplementary file 3 — Supplementary file3 (JPG 19955 kb) [file 12565_2025_851_MOESM3_ESM.jpg]

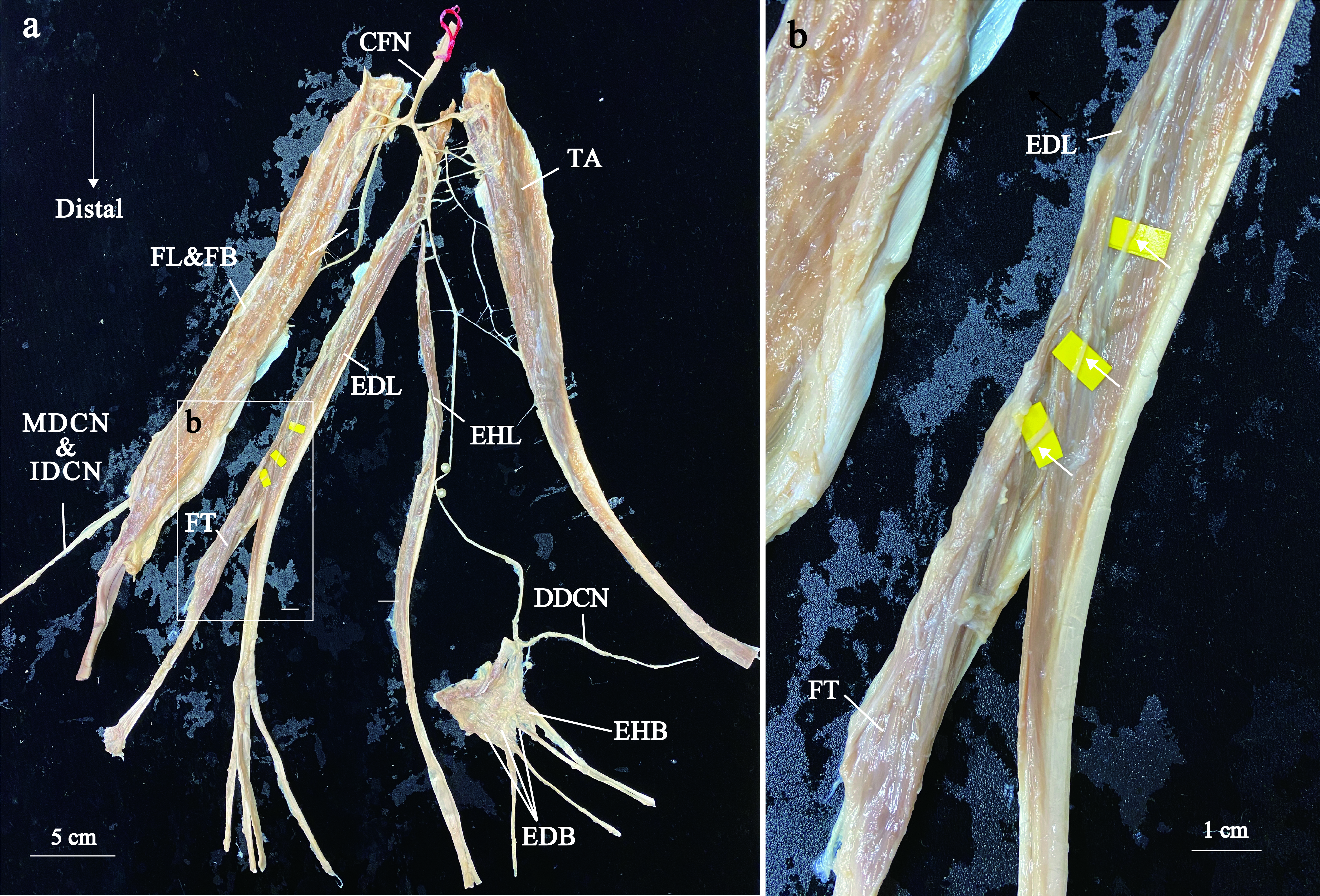

Supplement: Supplementary file 4 — Supplementary file4 (JPG 23051 kb) [file 12565_2025_851_MOESM4_ESM.jpg]

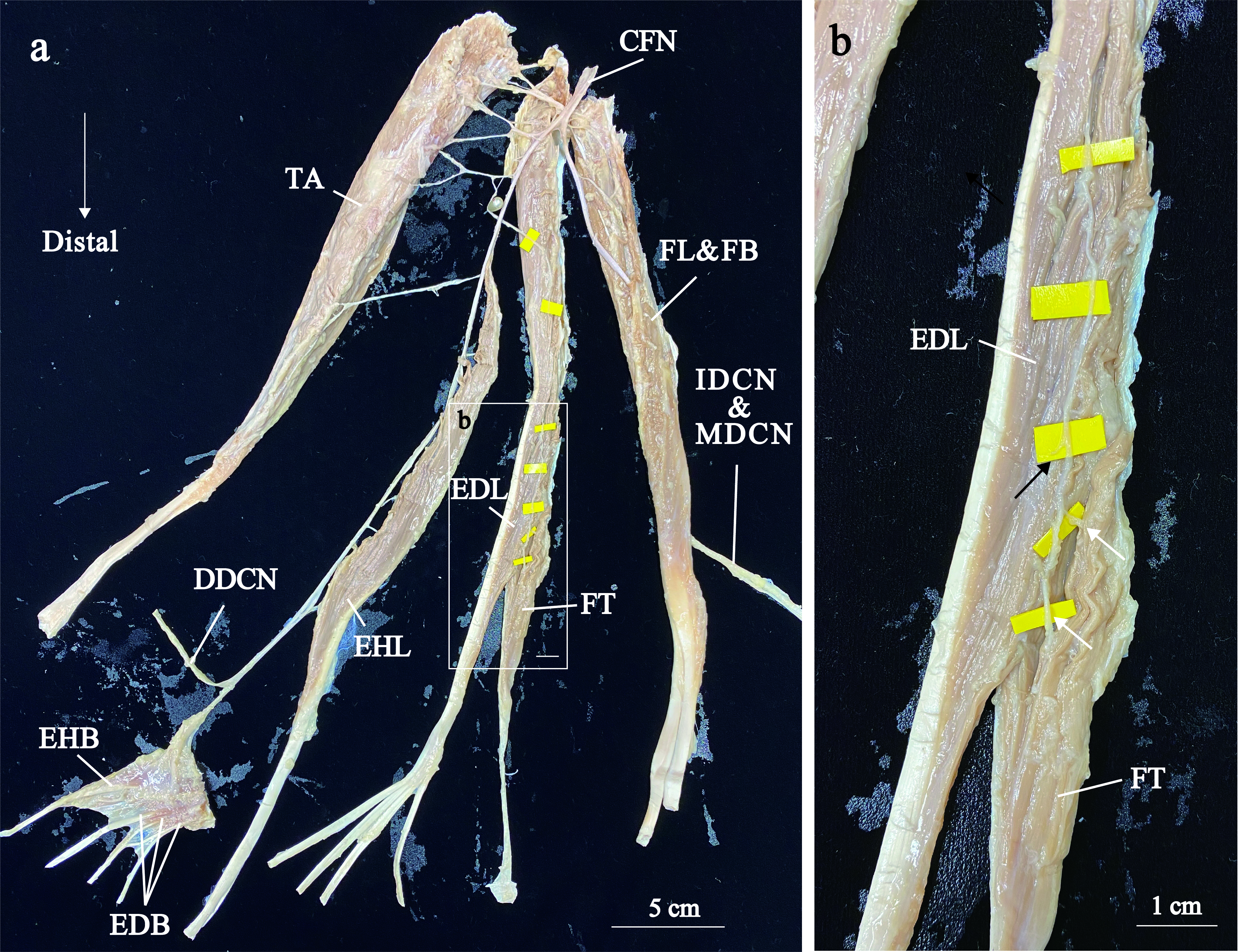

Supplement: Supplementary file 5 — Supplementary file5 (JPG 18834 kb) [file 12565_2025_851_MOESM5_ESM.jpg]

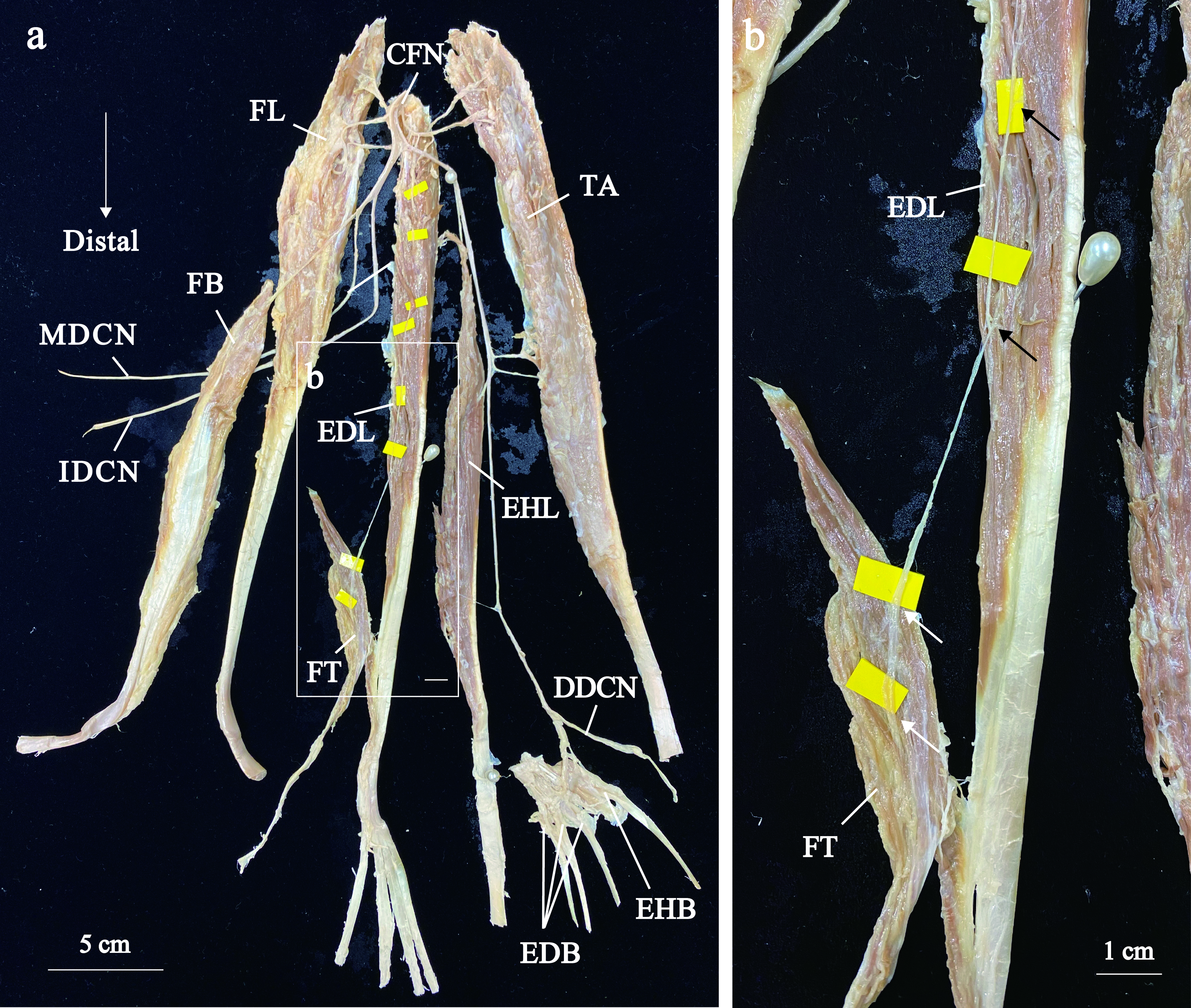

Supplement: Supplementary file 6 — Supplementary file6 (JPG 18279 kb) [file 12565_2025_851_MOESM6_ESM.jpg]

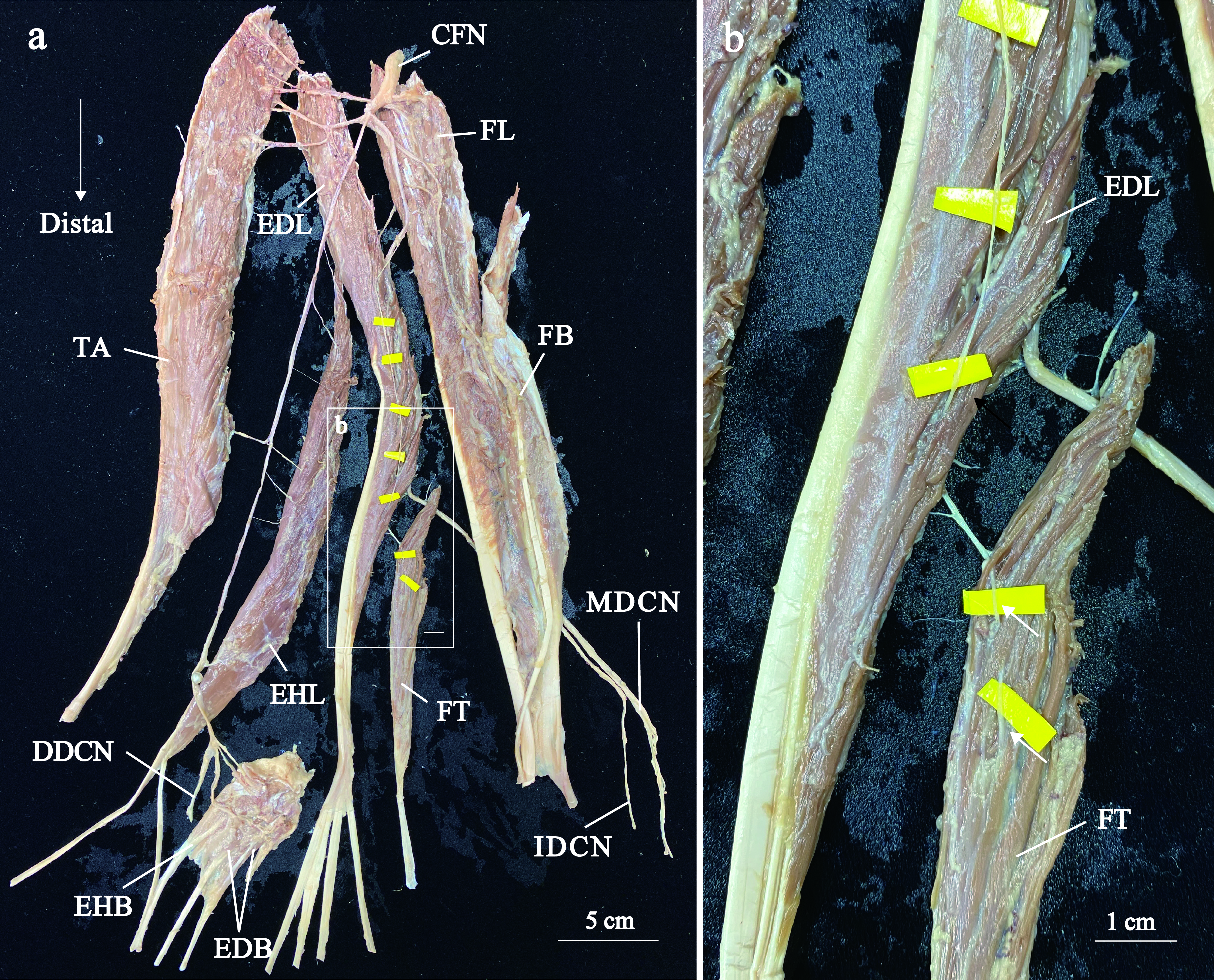

Supplement: Supplementary file 7 — Supplementary file7 (JPG 21704 kb) [file 12565_2025_851_MOESM7_ESM.jpg]

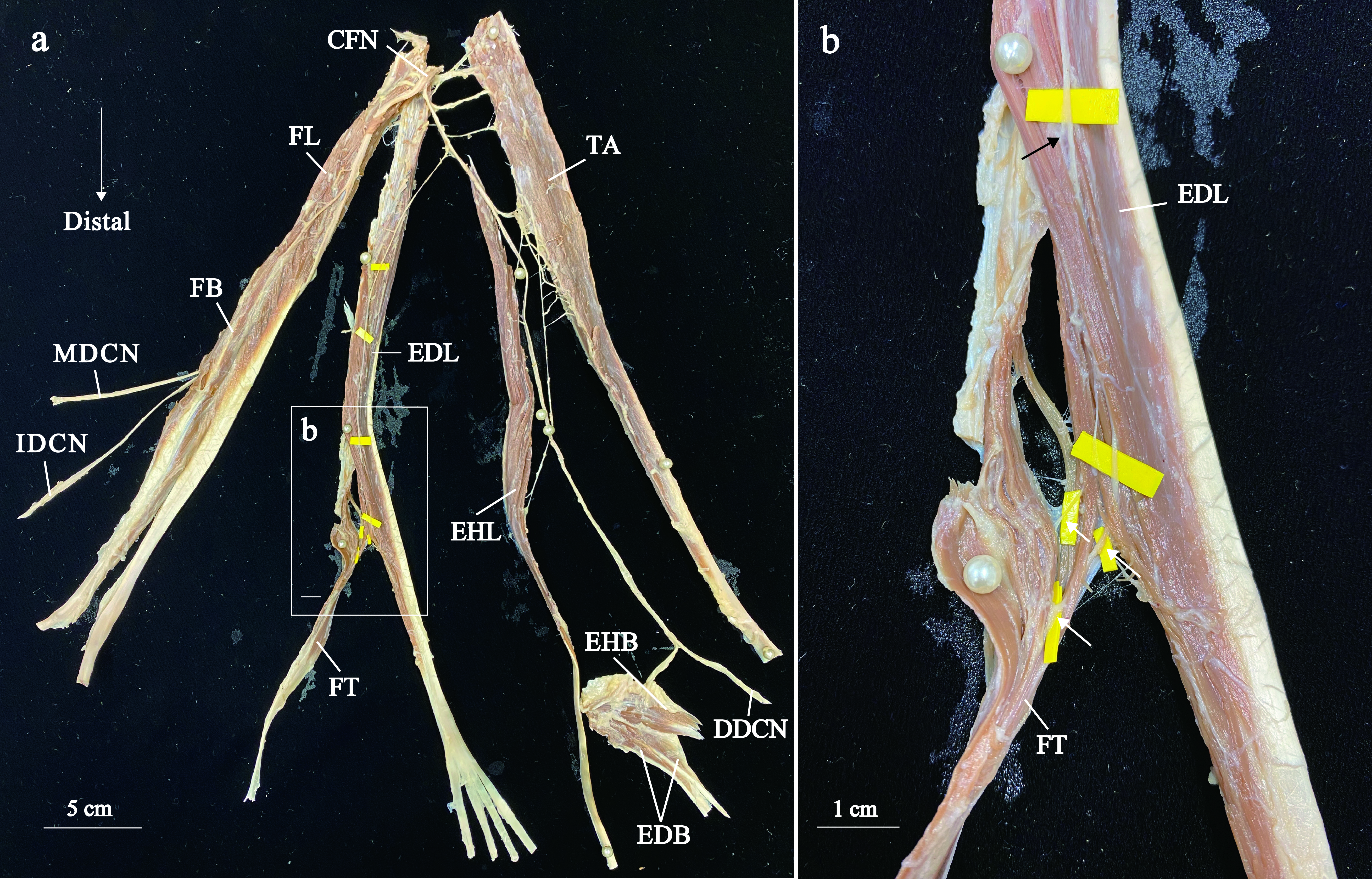

Supplement: Supplementary file 8 — Supplementary file8 (JPG 23288 kb) [file 12565_2025_851_MOESM8_ESM.jpg]

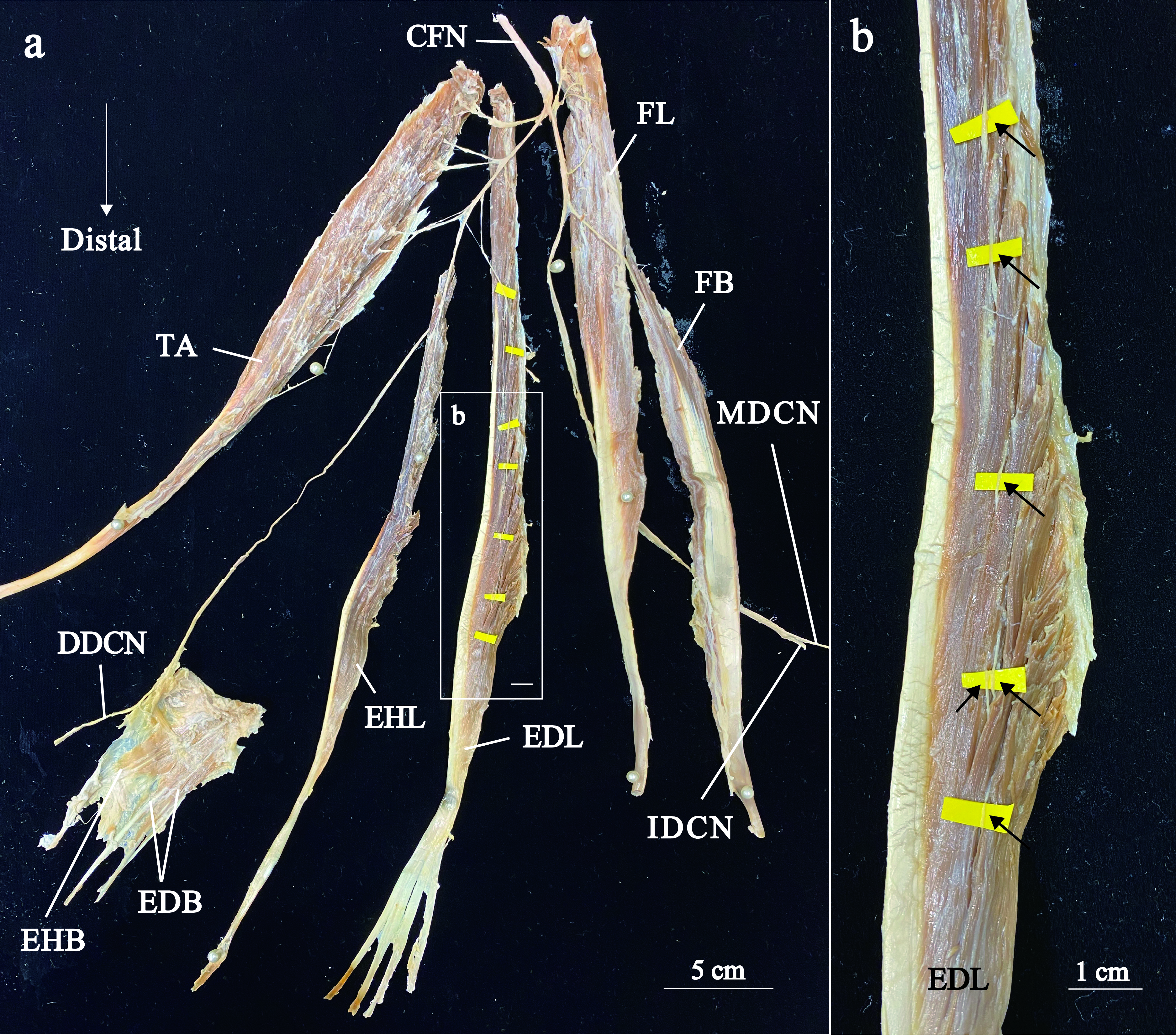

Supplement: Supplementary file 9 — Supplementary file9 (JPG 16881 kb) [file 12565_2025_851_MOESM9_ESM.jpg]

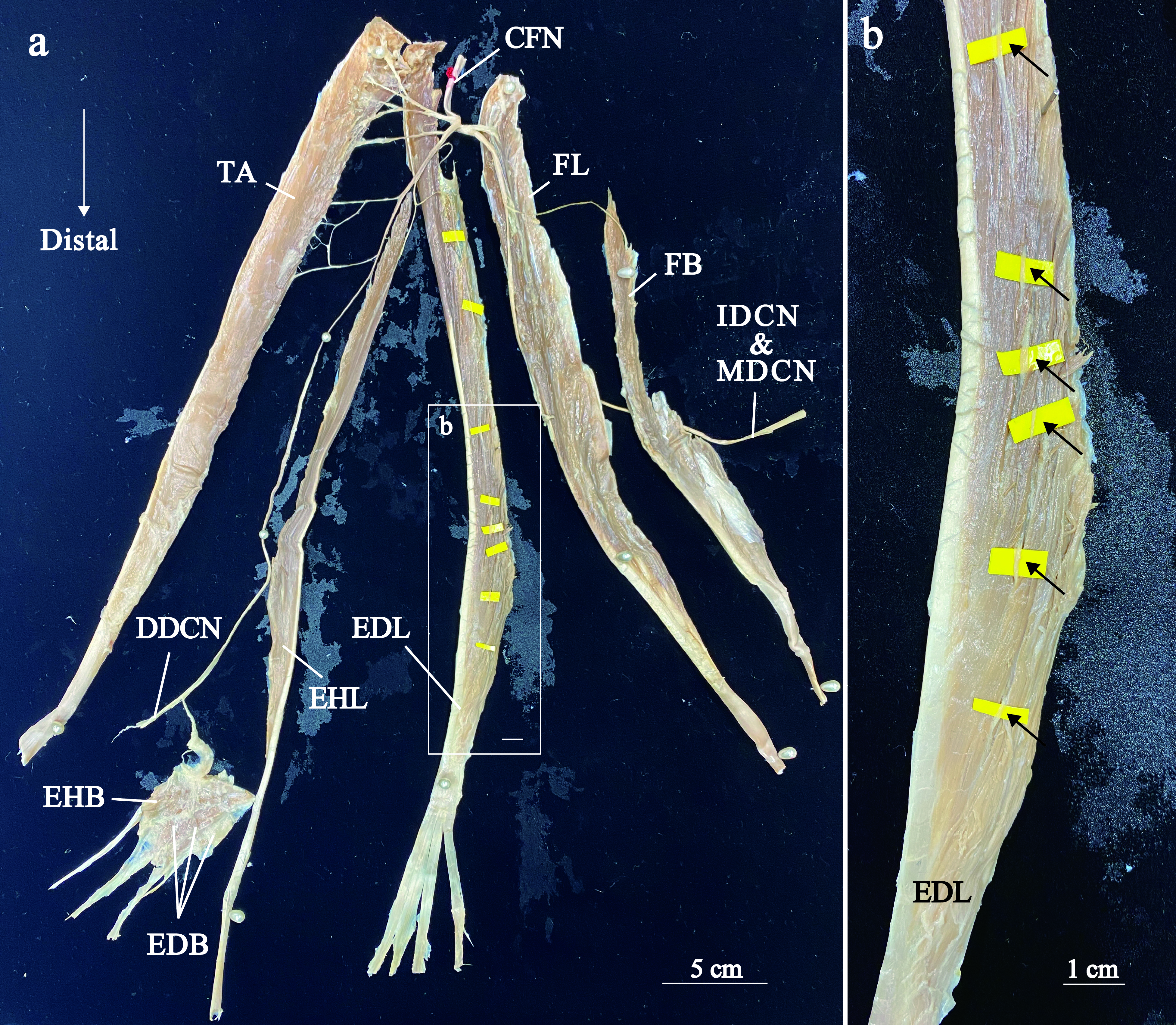

Supplement: Supplementary file 10 — Supplementary file10 (JPG 18935 kb) [file 12565_2025_851_MOESM10_ESM.jpg]

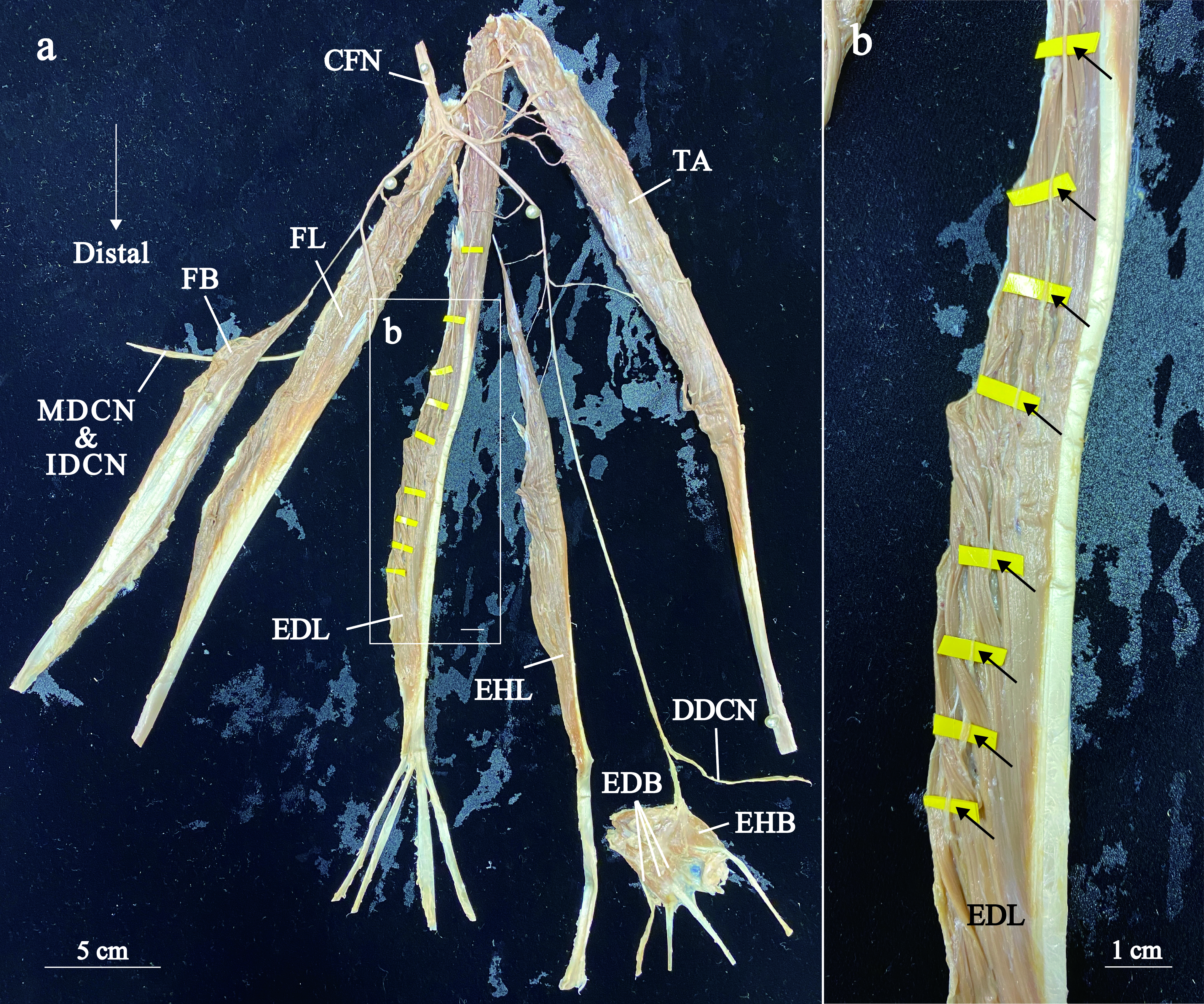

Supplement: Supplementary file 11 — Supplementary file11 (JPG 20120 kb) [file 12565_2025_851_MOESM11_ESM.jpg]
